# The Potential Mechanisms of Catechins in Tea for Anti-Hypertension: An Integration of Network Pharmacology, Molecular Docking, and Molecular Dynamics Simulation

**DOI:** 10.3390/foods13172685

**Published:** 2024-08-26

**Authors:** Yanming Tuo, Xiaofeng Lu, Fang Tao, Marat Tukhvatshin, Fumin Xiang, Xi Wang, Yutao Shi, Jinke Lin, Yunfei Hu

**Affiliations:** 1 College of Horticulture, Fujian Agriculture and Forestry University, Fuzhou 350002, China; tuo3152022@163.com (Y.T.); lxfitea21@163.com (X.L.); whitehydrangea@163.com (F.T.); marattukhvatshin@mail.ru (M.T.); fuminrz@163.com (F.X.); wx553829422@163.com (X.W.); ytshi@wuyiu.edu.cn (Y.S.); 2 College of Tea and Food Sciences, Wuyi University, Wuyishan 354300, China; 3 Anxi College of Tea Science, Fujian Agriculture and Forestry University, Fuzhou 350002, China

**Keywords:** tea, catechins, hypertension, network pharmacology, molecular docking, molecular dynamics simulation

## Abstract

Catechins, a class of polyphenolic compounds found in tea, have attracted significant attention due to their numerous health benefits, particularly for the treatment and protection of hypertension. However, the potential targets and mechanisms of action of catechins in combating hypertension remain unclear. This study systematically investigates the anti-hypertensive mechanisms of tea catechins using network pharmacology, molecular docking, and molecular dynamics simulation techniques. The results indicate that 23 potential anti-hypertensive targets for eight catechin components were predicted through public databases. The analysis of protein–protein interaction (PPI) identified three key targets (MMP9, BCL2, and HIF1A). KEGG pathway and GO enrichment analyses revealed that these key targets play significant roles in regulating vascular smooth muscle contraction, promoting angiogenesis, and mediating vascular endothelial growth factor receptor signaling. The molecular docking results demonstrate that the key targets (MMP9, BCL2, and HIF1A) effectively bind with catechin components (CG, GCG, ECG, and EGCG) through hydrogen bonds and hydrophobic interactions. Molecular dynamics simulations further confirmed the stability of the binding between catechins and the targets. This study systematically elucidates the potential mechanisms by which tea catechins treat anti-hypertension and provides a theoretical basis for the development and application of tea catechins as functional additives for the prevention of hypertension.

## 1. Introduction

Tea is one of the three major non-alcoholic beverages consumed worldwide. Tea polyphenols, important natural components found within tea, possess various health benefits [1,2]. Among these, catechins constitute the primary portion of tea polyphenols, accounting for approximately 80% of the total. The main catechins include catechin gallate (CG), epicatechin gallate (ECG), gallocatechin gallate (GCG), epigallocatechin gallate (EGCG), catechin (C), epicatechin (EC), gallocatechin (GC), and epigallocatechin (EGC) [3,4]. Catechins are crucial components responsible for the numerous health benefits conferred by tea. Research has shown that catechins possess potential health benefits in antioxidation, anti-hypertension, and hypoglycemia, thus offering new possibilities for hypertension (HTN) treatment and prevention [5,6,7,8].

The long-term condition of hypertension has emerged as a critical global healthcare issue, presenting a significant challenge in the field of public health [9]. According to the World Health Organization (WHO), the number of individuals with hypertension is expected to reach approximately 1.56 billion by 2025 [10]. Hypertension, while not the primary cause of mortality, can lead to complications such as cardiovascular disease, heart failure, and renal failure [11,12]. Hypertensive patients, who experience prolonged elevated blood pressure, endure increased pressure on the vascular walls, leading to conditions such as atherosclerosis and vascular sclerosis, which in turn result in vascular narrowing, blockage, or rupture [13]. Thus, there is an urgent need to develop effective treatment and prevention strategies to address this global health problem.

In recent years, natural products have become a focus of hypertension treatment research due to their lower side effects and higher safety profiles. Tea catechins, as significant natural products, have shown potential anti-hypertensive effects. Studies have indicated that improving arterial elasticity is another mechanism for preventing hypertension. An epidemiological study with a cross-sectional design, which included 6589 adults aged 40–75 years in Wuyishan, Fujian Province, China, suggested that habitual tea drinking may protect against arterial stiffness [14]. Additionally, the consumption of green and black tea has been associated with reduced risks of cardiovascular disease and certain cancers [15]. These health benefits are typically attributed to the polyphenolic compounds in tea [16]. Garcia et al. [17] investigated the effects of green tea on blood pressure and sympathetic nerve excitation in an L-NAME-induced hypertensive rat model. The results showed that L-NAME-treated rats exhibited elevated blood pressure (165 mmHg) compared to control rats (103 mmHg), while green tea treatment reduced hypertension (119 mmHg). In vivo experiments have demonstrated the anti-hypertensive effects of a catechin-rich diet in rats [18]. Catechins may alleviate vascular dysfunction in hypertensive mice by regulating oxidative stress and endothelial nitric oxide synthase (eNOS) [19].

Despite the potential anti-hypertensive effects of tea catechins, further research is needed to explore their active components, mechanisms of action, and targets in hypertension treatment. To gain a deeper understanding of the pharmacological mechanisms by which drugs treat diseases, researchers have increasingly employed various computational methods, including network pharmacology, molecular docking, and molecular dynamics simulation [20]. Network pharmacology, an emerging research approach, integrates systems biology, network analysis, and pharmacology and is frequently employed to investigate the multi-target effects of complex natural compounds [21]. Molecular docking is a computational simulation method used to predict the binding modes and affinities between small molecules (drugs) and protein receptors (targets) [22]. Molecular dynamics simulation further verifies and optimizes the binding modes between these small molecules and protein receptors, providing a more precise molecular-level understanding [23].

**Figure 1:**
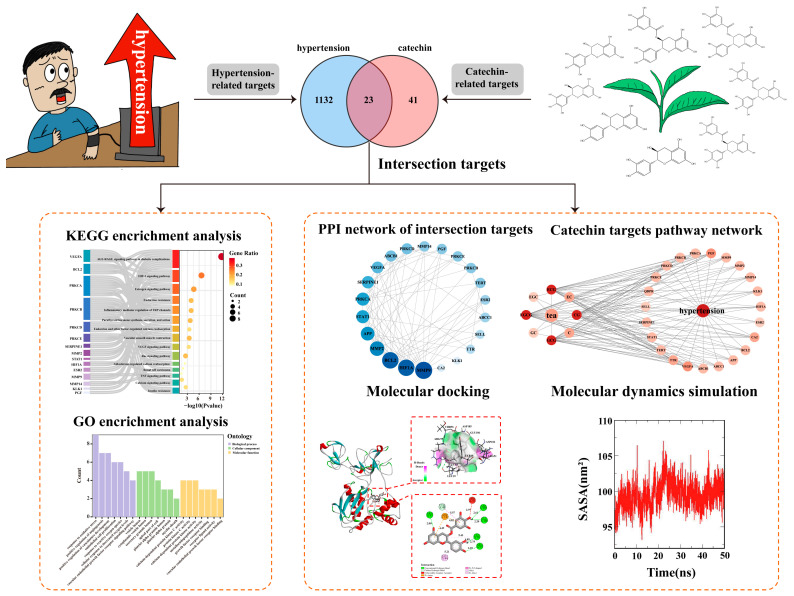
Network pharmacology regulatory mechanisms of catechins in anti-hypertension.

In this study, we aim to comprehensively elucidate the anti-hypertensive potential of tea catechin components, identify their targets, and explore their mechanisms of action using a combined approach of network pharmacology, molecular docking, and molecular dynamics simulation. This research will provide a theoretical basis for the further application of tea catechins in the biomedical field. The complete research process is illustrated in Figure 1.

## 2. Materials and Methods

### 2.1. Collection of Catechin Targets

**Figure 2:**
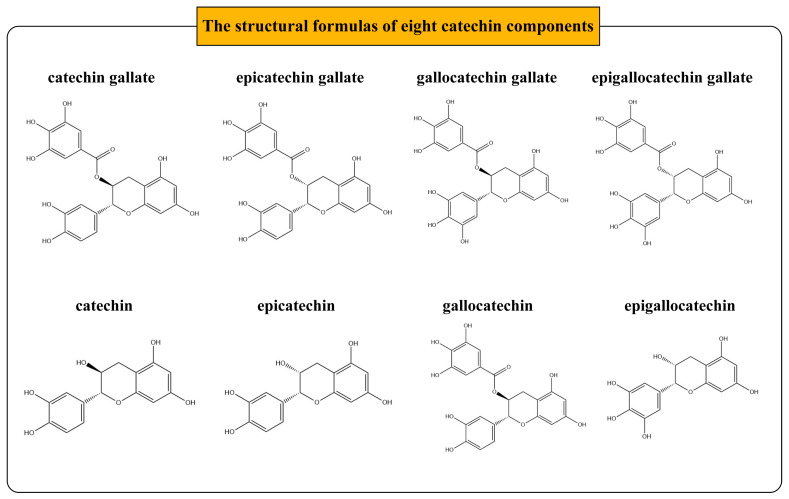
Structural formulas of eight catechin components.

The isomeric SMILES numbers for the eight catechin components—catechin (C), epicatechin (EC), gallocatechin (GC), epigallocatechin (EGC), catechin gallate (CG), epicatechin gallate (ECG), gallocatechin gallate (GCG), and epigallocatechin gallate (EGCG)—were obtained from the PubChem database (https://pubchem.ncbi.nlm.nih.gov/, accessed on 14 July 2024), with their structures illustrated in Figure 2. Subsequently, the predicted targets of catechins were identified using the isomeric SMILES numbers through the SEA Search Server (https://sea.bkslab.org/, accessed on 14 July 2024) and the SwissTargetPrediction (http://www.swisstargetprediction.ch/, accessed on 14 July 2024) databases. The predicted targets from the two databases were then merged, and duplicates were removed, resulting in the collection of potential targets for the eight catechin components. Furthermore, the catechin–targets network was constructed and visualized using Cytoscape 3.9.1.

### 2.2. Screening of Hypertension-Related Targets

Hypertension-related targets were retrieved from three disease databases: DisGeNET (https://www.disgenet.org/, accessed on 15 July 2024), OMIM (https://omim.org/, accessed on 15 July 2024), and GeneCards (https://www.genecards.org/, accessed on 15 July 2024), using the keyword “hypertension”. The screening criteria were set as a score of gda ≥ 0.1 in the DisGeNET database and a relevance score ≥ 5 in the GeneCards database. The hypertension-related targets from the three databases were then merged, and duplicate targets were removed.

### 2.3. Screening of Key Targets and Construction and Analysis of the Protein–Protein Interaction (PPI) Network

To explore the potential targets of catechins in treating hypertension, an intersection analysis was performed between the catechin targets and hypertension targets, and a Venn diagram was generated using the online platform (https://cloud.metware.cn/, accessed on 21 July 2024). The intersecting targets were imported into the STRING database (http://string-db.org/, accessed on 21 July 2024) to construct the PPI network of the target proteins [24]. “Homo sapiens” was selected as the species, and the minimum interaction score threshold was set to 0.4, with other parameters set to default. The network was visualized using Cytoscape 3.9.1 software, and the Network Analysis plugin was employed to calculate the degree value of each target. Key targets were identified based on their degree values, and molecular docking studies were subsequently conducted on the top three key proteins with the highest degree values.

### 2.4. GO and KEGG Pathway Enrichment Analyses

To further investigate the biological functions of the key target genes, GO and KEGG enrichment analyses were performed using the “clusterProfiler” package, with a *p*-value threshold set at *p* < 0.05. GO functional analysis predicted gene functions across three categories: biological process (BP), cellular component (CC), and molecular function (MF), while KEGG pathway enrichment analysis identified key pathways associated with the anti-hypertensive targets of catechins. Both GO and KEGG analysis results were visualized using an online tool (https://cloud.metware.cn/, accessed on 21 July 2024).

### 2.5. Molecular Docking

The SDF format ligand files for the eight catechin components were obtained from the PubChem database (https://pubchem.ncbi.nlm.nih.gov/, accessed on 23 July 2024), and the three-dimensional structure models of the proteins MMP9 (PDB ID: 1L6J), HIF1A (PDB ID: 4H6J), and BCL2 (PDB ID: 5FCG) were obtained from the UniProt database (https://www.uniprot.org/, accessed on 23 July 2024) [25]. Molecular docking was conducted using AutoDock Vina 1.2.5 [26], and the docking results were further analyzed and visualized using Discovery Studio 2019 software.

### 2.6. Molecular Dynamics Simulation

To explore the stability of the protein–ligand interactions in greater depth, molecular dynamics (MD) simulations were performed on four complexes: CG-MMP9, GCG-MMP9, ECG-HIF1A, and EGCG-BCL2, using GROMACS 2020.6 software. The AMBER99SB force field and SPC water model were utilized, with the system temperature set at 300 K and the simulation time at 50 ns. The energy minimization phase employed the steepest descent method, followed by energy equilibration to stabilize the system before completing the MD simulation. The binding free energies were calculated using the MM/GBSA method, and the resulting MD simulation data were visualized using Xmgrace software 5.1.25 [27].

## 3. Results

### 3.1. Screening of Potential Targets for the Anti-Hypertensive Effects of Catechin Components

**Figure 3:**
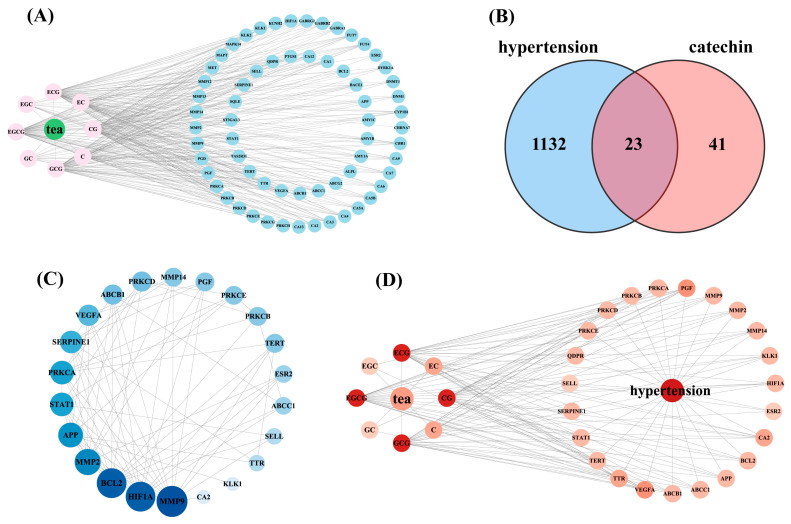
(**A**) Construction of the tea–catechin–targets network. (**B**) Venn diagram of predicted catechin targets and hypertension-related targets. (**C**) PPI network of intersecting targets. (**D**) tea–catechin–targets–hypertension network diagram.

For the eight catechin components, 51 and 48 targets were predicted from the SEA Search Server and Swiss Target Prediction databases, respectively. After removing duplicate targets, 64 unique targets for the catechin components were identified, and a tea–catechin–targets network was constructed using Cytoscape 3.9.1 (Figure 3A, Supplementary Table S1). Among these, ECG had the most associated potential targets (61), followed by EGCG (60), GCG (60), CG (59), C (24), EC (24), GC (13), and EGC (13). Additionally, hypertension-related targets were gathered from multiple databases, resulting in 784 targets from DisGeNET, 683 from GeneCards, and 75 from OMIM. After removing duplicates, 1155 hypertension-related targets were obtained (Supplementary Table S2). A Venn diagram was used to identify the intersecting targets between the predicted catechin targets and hypertension targets. The analysis identified 23 targets as potential candidates for the anti-hypertensive action of catechins (Figure 3B).

### 3.2. Protein–Protein Interaction (PPI) Network Analysis and Key Target Screening

The PPI network for the 23 intersecting targets was constructed using the STRING database and visualized with Cytoscape 3.9.1 software (Figure 3C). The PPI network comprised 22 nodes and 150 edges, with node size and color varying according to their degree value (Supplementary Table S3). The degree value represents the number of edges connected to a node, indicating its importance. Among the targets, MMP9, BCL2, and HIF1A exhibited the highest degree values, with degrees of 15, 14, and 14, respectively, identifying them as potential key targets.

To determine the key catechin components for anti-hypertension, an interaction network between the eight catechin components and the intersecting targets was constructed (Figure 3D). Notably, all these catechin components interacted with multiple targets. Among the eight catechin components, GCG, EGCG, ECG, and CG had the highest degree values (degree = 22), followed by C and EC (degree = 7) and, lastly, CG and EGC (degree = 3) (Supplementary Table S4). In summary, GCG, EGCG, ECG, and CG are likely the essential catechin components for combating hypertension.

### 3.3. Functional Enrichment Analysis of Key Targets

**Figure 4:**
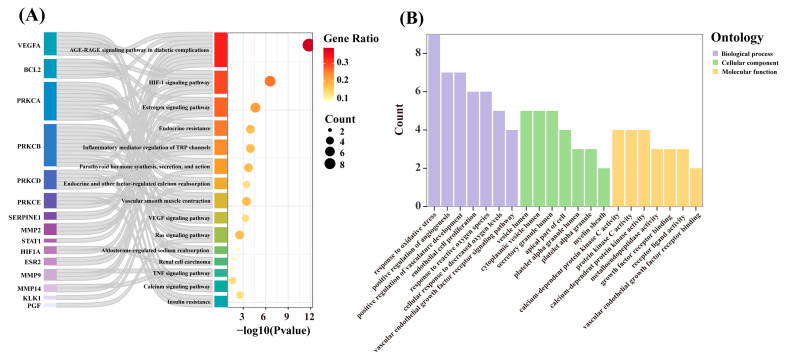
(**A**) KEGG pathway analysis of key targets. (**B**) GO enrichment analysis of key targets.

To comprehensively understand the mechanisms by which catechin components act on hypertension at the system level, KEGG and GO enrichment analyses were performed on the 23 intersecting targets. KEGG enrichment analysis revealed that the 23 intersecting targets were enriched in 90 signaling pathways (*p* < 0.05, Supplementary Table S5), including the vascular smooth muscle contraction (hsa04270), aldosterone-regulated sodium reabsorption (hsa04960), renal cell carcinoma (hsa05211), and TNF signaling pathway (hsa04668), among others (Figure 4A). Additionally, the GO enrichment results show that the targets were enriched in 1063 biological processes (BPs), seven cellular components (CCs), and 89 molecular functions (MFs) (Supplementary Table S6, *p* < 0.05). As illustrated in Figure 4B, in the BP category, the main enrichments were in positive regulation of angiogenesis (GO:0045766), endothelial cell proliferation (GO:0001935), and vascular endothelial growth factor receptor signaling pathway (GO:0048010). In the CC category, the primary enrichments were in platelet alpha granule lumen (GO:0031093), platelet alpha granule (GO:0031091), and apical part of cell (GO:0045177). In the MF category, significant enrichments included calcium-dependent protein kinase C activity (GO:0010857), vascular endothelial growth factor receptor binding (GO:0005172), and metalloendopeptidase activity (GO:0004222). The results of the KEGG pathway and GO enrichment analyses suggest that the key targets play important roles in regulating vascular smooth muscle contraction, promoting angiogenesis, and mediating vascular endothelial growth factor receptor signaling processes.

### 3.4. Molecular Docking Verification

Molecular docking was employed to evaluate the binding affinities of four catechin components with three key target genes (MMP9, HIF1A, and BCL2). The results show that the docking scores for the four catechin components with the three targets ranged from −6.2 kcal/mol to −8.9 kcal/mol (Table 1). Lower docking scores indicate stronger binding affinities, with scores < −5.0 kcal/mol indicating potential binding and scores < −7.0 kcal/mol denoting strong binding affinities [28]. For MMP9, CG and GCG exhibited the strongest binding affinities, both scoring −8.9 kcal/mol. For HIF1A and BCL2, the strongest binding ligands were ECG and EGCG, with docking scores of −8.8 kcal/mol and −6.7 kcal/mol, respectively. The reference drug enalapril had docking scores of −7.1 kcal/mol, −6.7 kcal/mol, and −6.0 kcal/mol with MMP9, HIF1A, and BCL2, respectively. Compared to the reference drug enalapril, these significant compounds demonstrated stronger binding affinities to the key targets.

**Figure 5:**
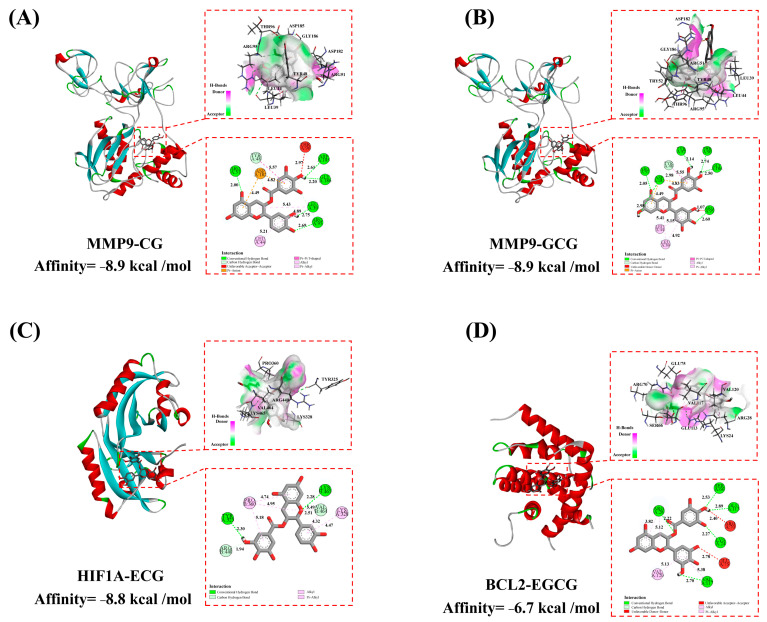
Molecular docking of main catechin components with MMP9, HIF1A, and BCL2. (**A**) Interaction diagram between MMP9 and gallocatechin gallate (GCG). (**B**) Interaction diagram between MMP9 and catechin gallate (CG). (**C**) Interaction diagram between HIF1A and epicatechin gallate (ECG). (**D**) Interaction diagram between BCL2 and epigallocatechin gallate (EGCG).

The binding mechanisms between the three key targets and their selected catechin ligands are shown in Figure 5. The binding pockets of MMP9, HIF1A, and BCL2 were tightly occupied by CG, GCG, ECG, and EGCG, stabilized by hydrogen bonds and hydrophobic interactions (Supplementary Table S7). In MMP9, CG formed hydrogen bonds with LEU39 (2.75 Å), ASP185 (2.61 Å), GLY186 (2.20 Å), ARG51 (2.00 Å), ARG95 (2.69 Å and 2.67 Å), and TYR48 (3.00 Å) and hydrophobic interactions with TYR48 (5.57 Å), LEU39 (5.43 Å and 4.89 Å), and LEU44 (5.21 Å) (Figure 5A). GCG in MMP9 formed hydrogen bonds with ASP182 (2.98 Å), THR96 (2.74 Å), GLY186 (2.50 Å), TYR52 (2.14 Å), ARG51 (2.05 Å), ARG95 (2.60 Å), and TYR48 (2.98 Å) and hydrophobic interactions with TYR48 (5.55 Å), LEU39 (5.41 Å and 4.92 Å), and LEU44 (5.15 Å) (Figure 5B). In HIF1A, ECG formed hydrogen bonds with TYR325 (2.30 Å), LYS465 (2.28 Å), ARG440 (1.94 Å), and VAL464 (2.51 Å) and hydrophobic interactions with PRO360 (4.95 Å), VAL464 (5.49 Å), PRO360 (4.74 Å), LYS328 (4.47 Å), VAL464 (4.32 Å), and PRO360 (5.18 Å) (Figure 5C). EGCG in BCL2 formed hydrogen bonds with VAL117 (2.70 Å), GLU113 (2.89 Å), LYS24 (2.27 Å), ARG28 (2.22 Å), and SER66 (2.53 Å) and hydrophobic interactions with ARG28 (5.12 Å and 3.82 Å), VAL117 (5.38 Å), and VAL120 (5.13 Å) (Figure 5D). These results indicate that catechin components effectively bind to the core targets through hydrogen bonds and hydrophobic interactions.

### 3.5. Molecular Dynamics Simulations and Binding Free Energy Calculations

**Figure 6:**
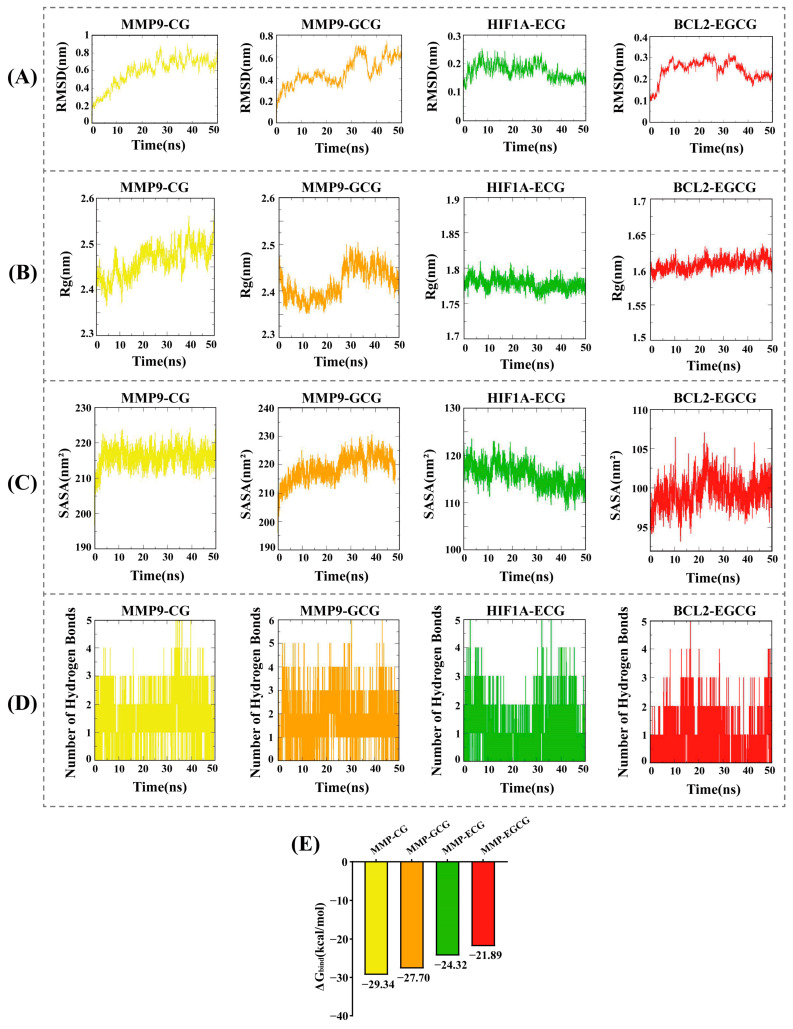
(**A**) RMSD values of the four complexes. (**B**) Radius of gyration (Rg) values of the four complexes. (**C**) Solvent-accessible surface area (SASA) values of the four complexes. (**D**) Number of hydrogen bonds in the four complexes. (**E**) Binding free energies (ΔG_bind_) of the four complexes.

To further explore the stability of protein–ligand interactions, molecular dynamics (MD) simulations were performed on four protein–ligand complexes: MMP9-CG, MMP9-GCG, HIF1A-ECG, and BCL2-EGCG. The root mean square deviation (RMSD) values were used to assess whether the simulation systems reached a stable state, with RMSD values within 1 nm indicating relative stability of protein–ligand interactions in a physiological environment [29]. As shown in Figure 6A, the RMSD values of the four complexes rapidly stabilized at 0.59 ± 0.16 Å, 0.46 ± 0.12 Å, 0.17 ± 0.03 Å, and 0.24 ± 0.05 Å, respectively. The radius of gyration (Rg) was analyzed to evaluate the compactness of receptor–ligand binding. As depicted in Figure 6B, the Rg values of the complexes remained stable throughout the simulation, stabilizing at 2.46 ± 0.03 nm, 2.42 ± 0.03 nm, 1.78 ± 0.01 nm, and 1.61 ± 0.01 nm, respectively. The solvent-accessible surface area (SASA) is an important parameter reflecting protein folding and stability. The SASA values of the four complexes also demonstrated stability, reaching average values of 215.79 ± 2.79 nm^2^, 218.87 ± 4.42 nm^2^, 115.76 ± 2.49 nm^2^, and 99.47 ± 1.90 nm^2^, respectively (Figure 6C). The number of hydrogen bonds reflects the strength of protein–ligand binding, with MMP9-GCG showing the highest hydrogen bond density and strength, followed by MMP9-CG, HIF1A-ECG, and BCL2-EGCG (Figure 6D).

The binding free energies (ΔG_bind_) of the four protein–ligand complexes were further calculated using the MM/GBSA method. Lower ΔG_bind_ values indicate stronger receptor–ligand binding affinity [30]. As shown in Figure 6E, the ΔG_bind_ rankings of the four complexes were MMP9-CG (−29.34 kcal/mol) < MMP9-GCG (−27.7 kcal/mol) < HIF1A-ECG (−24.32 kcal/mol) < BCL2-EGCG (−21.89 kcal/mol), which is consistent with the molecular docking results.

## 4. Discussion

Hypertension is increasingly acknowledged as a critical global public health concern due to its chronic nature [9]. Catechins have been confirmed to resist atherosclerosis, regulate intestinal microbiota dysbiosis, improve endothelial cell function, and modulate inflammatory signaling, thereby mitigating the adverse effects of hypertension [18,31]. Network pharmacology, known for its “multi-component, multi-target, and multi-pathway” characteristics, is utilized in this study to investigate the primary targets and potential mechanisms by which catechins exert anti-hypertensive effects, integrating it with computational simulation techniques.

The tea catechin–targets–hypertension network indicates that GCG, EGCG, ECG, and CG have high degree values. Consequently, these key catechin components were further analyzed for their therapeutic effects on hypertension. Chronic inflammation is considered a significant cause of hypertension, and reducing inflammation may aid in the prevention and treatment of hypertension [32]. It has been reported that EGCG, GCG, and CG possess various biological and pharmacological activities, including antioxidant, anti-inflammatory, and anti-angiogenic properties [33,34,35]. ECG and EGCG in tea play crucial roles in reducing serum cholesterol levels and inhibiting postprandial hyperlipidemia [36]. Redford et al. [37] found through rat experiments that the activation of vascular and neuronal KCNQ5 potassium channels significantly promotes vasodilation in both green and black tea, suggesting that ECG, EGCG, or their optimized derivatives are potential candidates for future anti-hypertensive drug development.

PPI network analysis revealed three potential key targets—MMP9, HIF1A, and BCL2—that are significantly implicated in the treatment of hypertension with catechins. These genes likely play a role in the therapeutic mechanisms by which catechins mitigate hypertension. Studies have shown that the knocking out of matrix metalloproteinase 9 (MMP9) in hypertensive rats can prevent the development of hypertension, proteinuria, glomerular damage, and renal interstitial fibrosis [38]. Cardiovascular diseases significantly impact blood pressure levels, and BCL2, a key anti-apoptotic protein, can serve as a target for modern cardioprotective therapies [39]. HIF1A, a hypoxia-inducible factor, was found to play an essential role in activating the transcriptional cofactor FOG2 in a CMVD mouse model of mild hypertension–diabetes injury, a condition relevant to human disease [40]. Therefore, MMP9, HIF1A, and BCL2 are closely linked to the onset and progression of hypertension, and catechin components may deliver their anti-hypertensive benefits through interactions with these target proteins.

GO and KEGG pathway enrichment analyses revealed key pathways involved in the anti-hypertension effects of catechins. The GO enrichment analysis indicated that the key targets of catechins were primarily enriched in biological processes such as the positive regulation of angiogenesis, the vascular endothelial growth factor receptor signaling pathway, and the vascular endothelial growth factor receptor binding. KEGG pathway enrichment analysis revealed that catechins were involved in several signaling pathways pertinent to hypertension treatment, including vascular smooth muscle contraction, aldosterone-regulated sodium reabsorption, and renal cell carcinoma. Vascular smooth muscle is a crucial component of the blood vessel wall; under hypertensive conditions, its sustained contraction leads to thickening of the vessel wall and increased vascular resistance, thereby elevating blood pressure [41]. Pulmonary arterial hypertension (PAH) is a progressive and complex pulmonary vascular disease, with HIF1A identified as a potential biomarker and therapeutic target for PAH [42]. Aldosterone, a hormone secreted by the adrenal glands, causes sodium and water retention, increasing blood volume and consequently raising blood pressure when at elevated levels, such as in primary aldosteronism [43]. Studies have shown that aldosterone induces MMP9 expression by activating CaMKII through oxidative stress. This exerts direct toxic effects on the myocardium, leading to increased cardiac rupture and mortality post-myocardial infarction in mice [44]. Renal cell carcinoma may contribute to hypertension by activating the renin–angiotensin system and affecting renal structure and function [45]. BCL2 and MMP9 have been found to play significant roles in tumor immunity and may serve as potential novel biomarkers and therapeutic targets for immunotherapy of clear cell renal cell carcinoma (ccRCC) [46,47]. Therefore, catechins achieve their anti-hypertensive effects through the regulation of various biological pathways.

Further, computational simulations were used to explore the binding affinity and mechanisms of catechins with key hypertension-related targets. The molecular docking results show that the docking scores for the four catechin components with the three targets were all less than −5 kcal/mol. The optimal catechin–ligand complexes formed with the three targets were MMP9-CG, MMP9-GCG, HIF1A-ECG, and BCL2-GCG, primarily driven by hydrogen bonding and hydrophobic interactions. Subsequently, molecular dynamics simulations were conducted to analyze the dynamic behavior and stability of the catechin–target complexes over time. The values of RMSD, Rg, and SASA and the number of hydrogen bonds indicated that the four complexes possessed reliable structural stability and compactness. Additionally, the binding free energies (ΔG_bind_) of the four protein–ligand complexes were analyzed using the MM/PBSA method, showing strong binding affinities, which corroborated the molecular docking results.

This study used network pharmacology to predict three key targets and pathways associated with the anti-hypertensive effects of four catechin components. Futhermore, the binding mechanisms between these catechin components and key targets were investigated using computer simulations. However, this study has certain limitations. Future research should utilize cell experiments and animal models to validate and explore the mechanisms by which catechins regulate hypertension-related pathways in vivo and in vitro, aiming to provide more effective and safer treatment options for patients with hypertension.

## 5. Conclusions

In this study, network pharmacology and computer simulation were used to explore the potential targets and molecular mechanisms of catechins in anti-hypertension. CG, GCG, ECG, and EGCG were identified as key catechin components for combating hypertension, with MMP9, HIF1A, and BCL2 as potential critical therapeutic targets. Molecular docking and molecular dynamics simulation results illustrate that catechins effectively bind to key targets through hydrogen bonding and hydrophobic interactions. These findings support the potential of catechins as functional additives for treating or preventing hypertension, providing a valuable foundation for further research. However, it is important to note that further pharmacological and clinical studies are required to validate our conclusions.

## Figures and Tables

**Table 1:** Molecular docking results of catechin components with core targets.

Compound Name	Affinity (kcal/mol)			
	MMP9	HIF1A	BCL2	Mean
CG	−8.9	−8.6	−6.5	−8.0
GCG	−8.9	−8.4	−6.5	−7.9
ECG	−8.0	−8.8	−6.2	−7.7
EGCG	−8.0	−8.5	−6.7	−7.7
Mean	−8.5	−8.6	−6.5	
enalapril	−7.1	−6.7	−6.0	−6.6

## Data Availability

The original contributions presented in the study are included in the article/supplementary material, further inquiries can be directed to the corresponding authors.

## Supplementary Materials

The following supporting information can be downloaded at: https://www.mdpi.com/article/10.3390/foods13172685/s1, Table S1: Information for 64 potential targets of catechin components; Table S2: Hypertension-related 1155 potential targets in 3 databases; Table S3: PPI network of key targets; Table S4: Catechin-core target degree; Table S5: 90 signaling pathways from KEGG enrichment analysis; Table S6: BP, CC, and MF from GO enrichment analysis; Table S7: Molecular docking data of four key catechin components and core.

## List of Major Abbreviations

Abbreviation	Full name
HTN	hypertension
C	catechin
EC	epicatechin
GC	gallocatechin
EGC	epigallocatechin
CG	catechin gallate
ECG	epicatechin gallate
GCG	gallocatechin gallate
EGCG	epigallocatechin gallate
RMSD	root mean square deviation
Rg	radius of gyration
SASA	solvent-accessible surface area
MD	molecular dynamics
BP	biological process
CC	cellular component
MF	molecular function
PPI	protein–protein interaction
ΔG_bind_	binding free energies
